# Supplementary material for: Molecularly specific detection towards trace nitrogen dioxide by utilizing Schottky-junction-based Gas Sensor
Source: Nat Commun. 2024 Jul 16;15:5991. doi: 10.1038/s41467-024-50443-5 (PMC11252297; doi:10.1038/s41467-024-50443-5)
Supplement: Supplementary file 1 — Supplementary information [file 41467_2024_50443_MOESM1_ESM.pdf]

Molecularly Specific Detection towards Trace Nitrogen Dioxide by Utilizing Schottky-Junction-based Gas Sensor

Shipu Xu[1, 2, 9*], Xuehan Zhou[3, 9], Shidang Xu[4, 9], Yan Zhang[5], Yiwen Shi[5], Xuzhong Cong[3], Qijia Xu[3], Ye Tian[6], Ying Jiang[6], Hanjie Guo[2], Jinkui Zhao[2, 7], Fengqiang Sun[5, 8*], Hailin Peng[3*].

[1]Songshan Lake Materials Laboratory

Dongguan 523808, P. R. China

[2]School of Microelectronics Science and Technology, Sun Yat-sen University

Zhuhai 519082, P. R. China

[3]Center for Nanochemistry, Beijing Science and Engineering Center for Nanocarbons, Beijing National Laboratory for Molecular Sciences, College of Chemistry and Molecular Engineering, Peking University

Beijing 100871, P. R. China

[4]School of Biomedical Sciences and Engineering, South China University of Technology

Guangzhou 511442, P. R. China

[5]School of Chemistry, South China Normal University

Guangzhou 510006, P. R. China

[6]International Center for Quantum Materials, School of Physics, Peking University

Beijing 100871, P. R. China

[7]The Institute of Physics, Chinese Academy of Sciences

Beijing 100190, P. R. China

[8]Key Laboratory of Theoretical Chemistry of Environment, Ministry of Education, South China Normal University

Guangzhou 510006, P. R. China

[9]These author contributed equally: Shipu Xu, Xuehan Zhou, Shidang Xu

*Corresponding author. Email: xushp7@mail.sysu.edu.cn (S.P. Xu); fqsun@scnu.edu.cn (F.Q. Sun); hlpeng@pku.edu.cn (H.L. Peng)

S1. The Epitaxial Growth of Bi_2O_2Se on the $SrTiO_3$ Substrate

Supplementary Figure 1a shows the X-ray diffraction (XRD) pattern of the Bi_2O_2Se grown on the $SrTiO_3$ (001) substrate. Towards out of plane, the $SrTiO_3$ substrate and the Bi_2O_2Se present lattice planes of (00l). The Bi_2O_2Se shows lattice planes of (002), (004), (006), (008), and (0010). Supplementary Fig. 1b is the scanning electron microscopy (SEM) image of the Bi_2O_2Se sheet, and it is etched to show the rectangle shape. This sample is applied for electron back scatter diffraction (EBSD) characterization. From the in-plane and the out-of-plane view, (001) lattice plane is observed for Bi_2O_2Se and $SrTiO_3$ (Supplementary Fig. 1c). The Bi_2O_2Se is demonstrated to be epitaxially grown along the (00l) crystal axis on the $SrTiO_3$ (001) substrate.

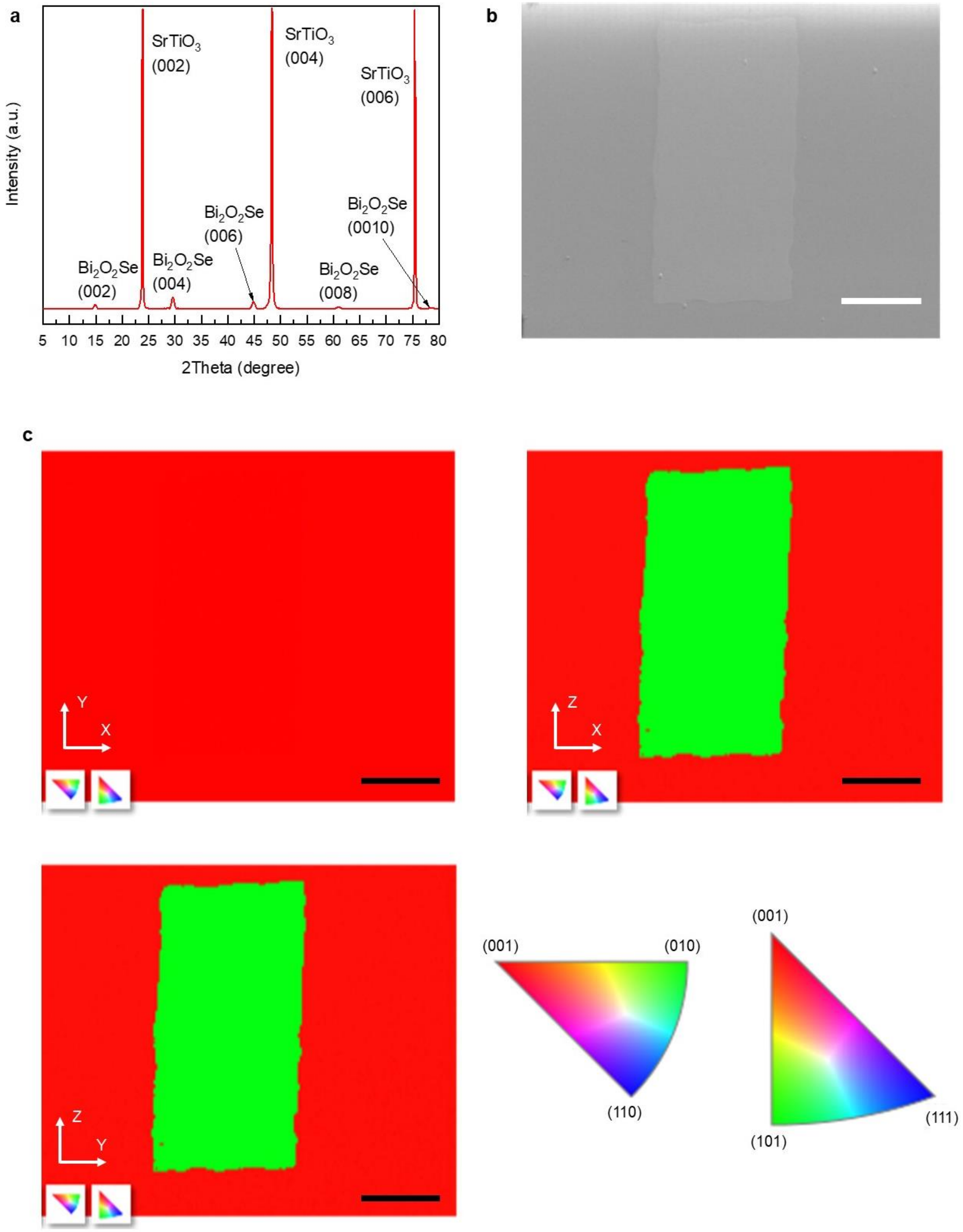

Supplementary Fig. 1| **a** XRD pattern of Bi_2O_2Se on the $SrTiO_3$ (001) substrate. **b** and **c** SEM image of the Bi_2O_2Se (**b**; scale bar: 10 μm) and its corresponding EBSD pattern from the in-plane and the out-of-plane view (**c**; scale bar: 10 μm).

S2. The Fermi Level of the Bi_2O_2Se

The ultraviolet photoelectron spectroscopy (UPS) is performed on the Bi_2O_2Se for

estimating its Fermi level. Supplementary Figure 2a shows the UPS of the Bi_2O_2Se that, its cutoff energy (E_{cutoff}) and Fermi energy (E_f) are respectively 15 eV and -1 eV. Then the Fermi level (E_F) of Bi_2O_2Se is calculated to be -5.2 eV according to the formular: $-E_F = E_p - (E_{cutoff} - E_f)$, where the E_p is the energy of excitation photoelectron and its value is 21.2 eV. Additionally, Supplementary Fig. 2b shows the X-ray photoelectron spectroscopy (XPS) spectrum of Bi_2O_2Se that it contains O 1s at 530 eV, Bi 4f7/2 and Bi 4f5/2 at 158 eV and 164 eV, Se 3d at 53 eV[1].

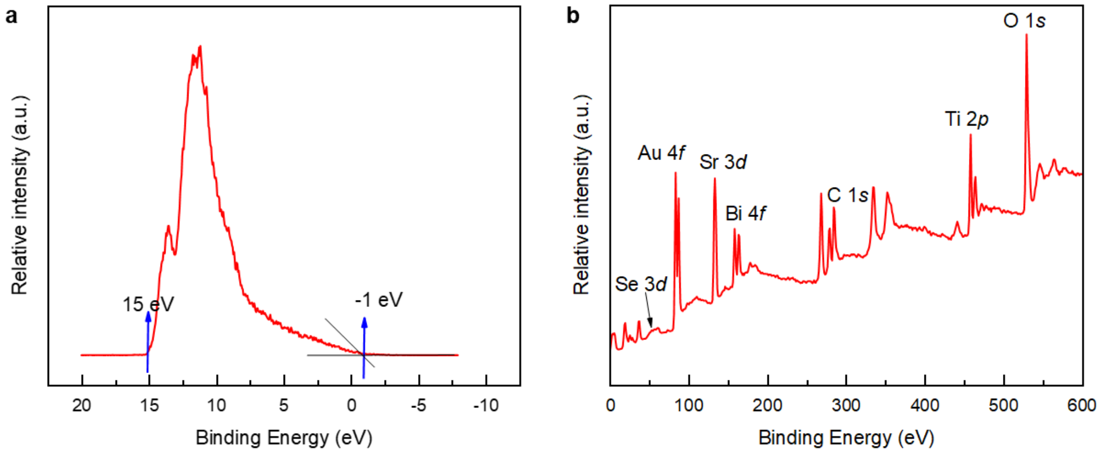

Supplementary Fig. 2| **a** and **b** The UPS (**a**) and the XPS pattern (**b**) of the Bi_2O_2Se.

S3. The Trivial Surface State of the Bi_2O_2Se

To reveal the trivial surface state, the Bi_2O_2Se is bonded with Pd/Au electrode to form a contact junction with a low barrier height, where the work function of Pd (5.1 eV) is lower than the Fermi level of Bi_2O_2Se (5.2 eV). By temperature (T) adjustment, the Bi_2O_2Se device is conducted with investigation about the current-voltage (I-V). In Supplementary Figure 3a, the current intensity is positively related to the voltage. By fitting $\ln(I/T^2)$ and $1/T$, the barrier height is estimated to be 0.085 eV[2, 3], indicating the trivial surface state induced by absorbates among the junction interface (Supplementary Fig. 3b).

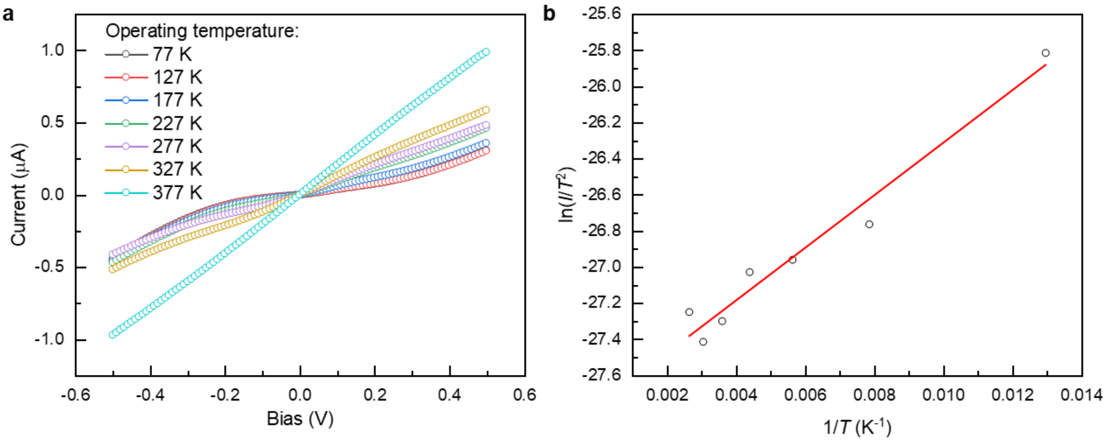

Supplementary Fig. 3| **a** and **b** *I*-*V* plot operated at different temperatures for the Bi_2O_2Se device (**a**; the Bi_2O_2Se bonded with the Pd/Au electrodes) and its linear fitting based on the thermionic emission model (**b**).

S4. The Bi_2O_2Se Hall Device

Supplementary Figure 4a shows the atomic force microscopy (AFM) image of the Bi_2O_2Se sheet, where its thickness is 7 nm and it is applied for the Hall device fabrication. Supplementary Fig. 4b presents the SEM image of the Bi_2O_2Se Hall device. The Bi_2O_2Se sheet is bonded with six electrodes for the Hall test. The electrode is consisted of 10-nm-thin Pd underneath and 90-nm-thin Au on the top. The *R*-*T* characterization is conducted on the Bi_2O_2Se device when the temperature increases from 10 K to 380K. Fig. S4c shows a positive relation between the Bi_2O_2Se resistance and temperature, indicating the metal-like feature of the Bi_2O_2Se.

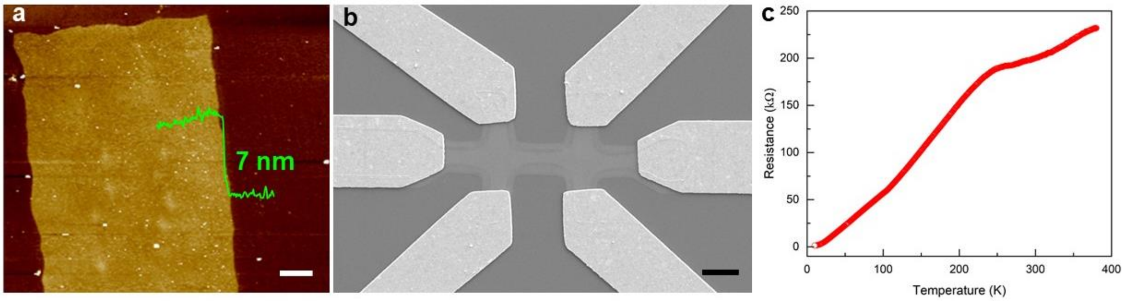

Supplementary Fig. 4| **a** The AFM image of the Bi_2O_2Se sheet (scale bar: 3 μm). **b** The SEM image of the Bi_2O_2Se device (scale bar: 2 μm). **c** *R*-*T* characterization for the Bi_2O_2Se device.

S5. The Investigation into the Diffusion Coefficients

The self-diffusion coefficient is defined as the dR/dt or dX/dt or dΘ/dt. The self-diffusion coefficient is relied on type of the adsorbate and its surface doping/scattering, and its Pearson's r in the linear fitting reflects the interference originated from the surficial behaviors[5, 6]. Supplementary Figure 5a shows the sensing signals to the target gas of acetone. The gas-concentration dependent self-diffusion coefficients are linearly fitted with the acetone concentration (Supplementary Fig. 5b). The Pearson's r for the reactance and the impedance is higher than that of the resistive. In the resistive sensing, the surface scattering is revealed to interfere the acetone doping.

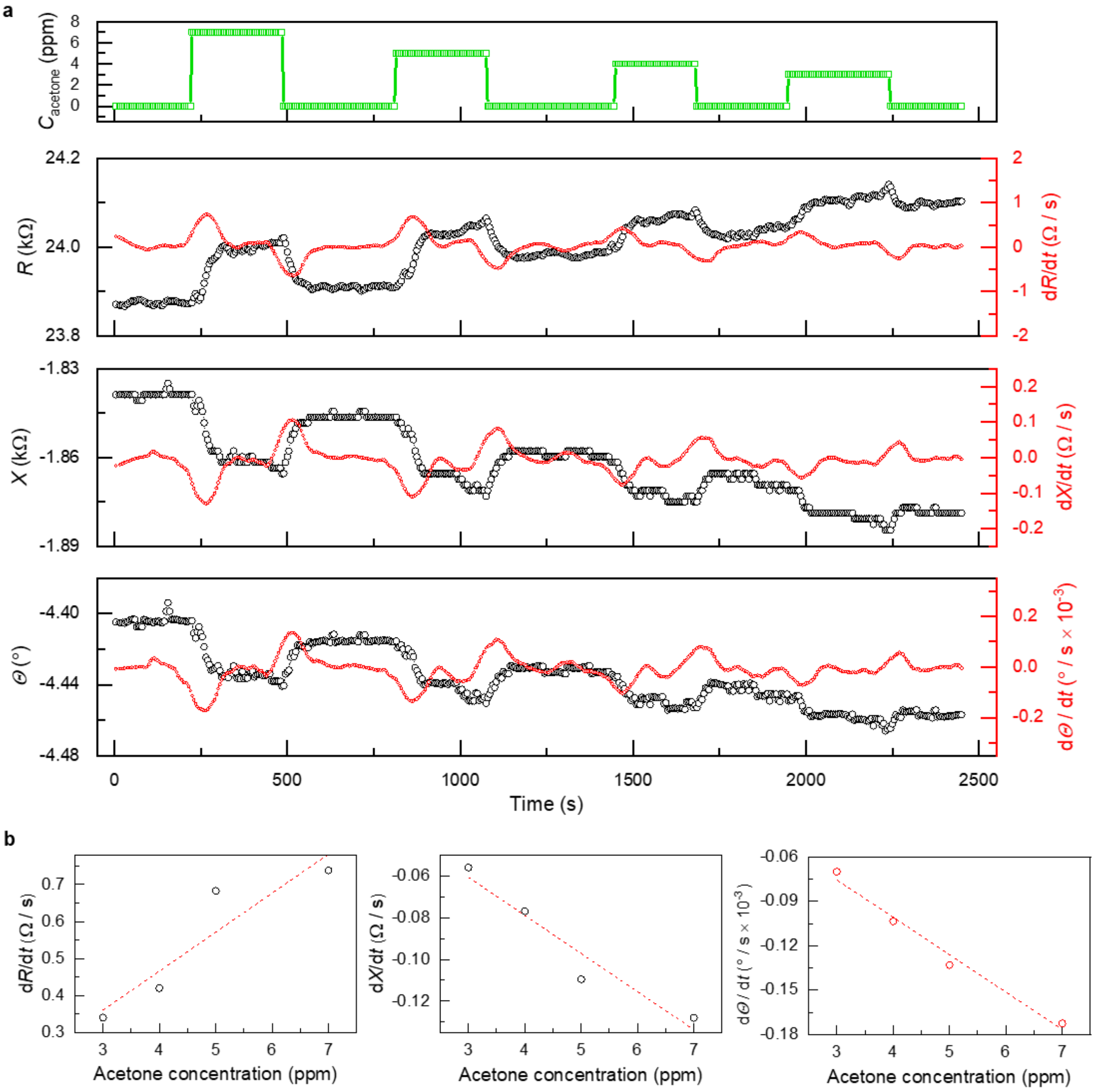

Supplementary Fig. 5| a Acetone-sensing signals of the resistive, reactance, and

impedance angle. **b** The diffusion coefficient as the function of the acetone concentration.

S6. The Surface-Scattering-Dominated Sensing Mechanism

Supplementary Figure 6 illustrates the surface-scattering-dominated sensing mechanism. The impedance angle is correlated with the change in dipole moment of the target molecule adsorbed ($\mathbf{P}_{ads.}$ - $\mathbf{P}_0$)/$\mathbf{P}_0$, where $\mathbf{P}_0$ and $\mathbf{P}_{ads.}$ are respectively dipole moments for the target gas and its adsorbed state. Firstly, the target molecule is adsorbed on the sensing block to induce two kinds of surficial phenomena, *i.e.*, surface scattering and adsorbate doping. The adsorbate doping varies the carrier density of the sensing block (n) to adjust its resistance (R). In this mean, the carrier-density-dominated sensing mechanism outputs the resistive signal but shows two issues: (1) the surface scattering usually interferes the resistive signal and degrades the sensitivity; (2) the molecular hybrid orbital energy cannot be distinguished based on the adsorbate doping at room temperature or the higher.

Herein, in the trace gas detection, the impedance angle is extracted as the sensing signal featured with molecular specificity. The reason for this fact is that the target molecule adsorbed forms as the scattering center. This scattering center is characterized by the charge quantity (σ), which is positively related to the change in the dipole moment of the target molecule ($\sigma \propto (\mathbf{P}_{ads.} - \mathbf{P}_0)/\mathbf{P}_0$, $0 \leqslant (\mathbf{P}_{ads.} - \mathbf{P}_0)/\mathbf{P}_0 \leqslant 1$). In details, the target molecule is physically adsorbed on the Bi_2O_2Se in the preliminary state, where its charge center is of charge quantity σ_0 and featured with dipole moment of $\mathbf{P}_0$. Next the surface doping occurs and indicates the change in dipole moment of the target molecule adsorbed ($\mathbf{P}_{ads.}$ - $\mathbf{P}_0$)/$\mathbf{P}_0$, where the adsorbed state of the molecule is of dipole moment of $\mathbf{P}_{ads.}$ This adsorption induces the charge variation for the target molecule $\sigma_{ind.}$, and its value is equal to $((\mathbf{P}_{ads.} - \mathbf{P}_0)/\mathbf{P}_0)\sigma_0$. Equivalent to the parallel-plate capacitance, the electric field $\mathbf{E}_0$ in the preliminary state turns into the adsorbed state $\mathbf{E}_{ads.}$, which is featured with the permittivity $\varepsilon_{r,ads.}$. This permittivity $\varepsilon_{r,ads.}$ is positively correlated to the charge of scattering center σ. Then the charge of scattering center σ is confirmed to be positively related to the

change in the dipole moment of the target molecule ($\sigma \propto (\mathbf{P}_{ads.} - \mathbf{P}_0)/\mathbf{P}_0$, $0 \leqslant (\mathbf{P}_{ads.} - \mathbf{P}_0)/\mathbf{P}_0 \leqslant 1$).

In the case of ionized impurity scattering, the carrier mobility is related to the charge quantity. Considering that the impedance angle is mainly dominated by the carrier mobility (μ) in the gas sensing, the impedance angle is then dependent on the change in dipole moment of the target molecule adsorbed and featured with molecular specificity.

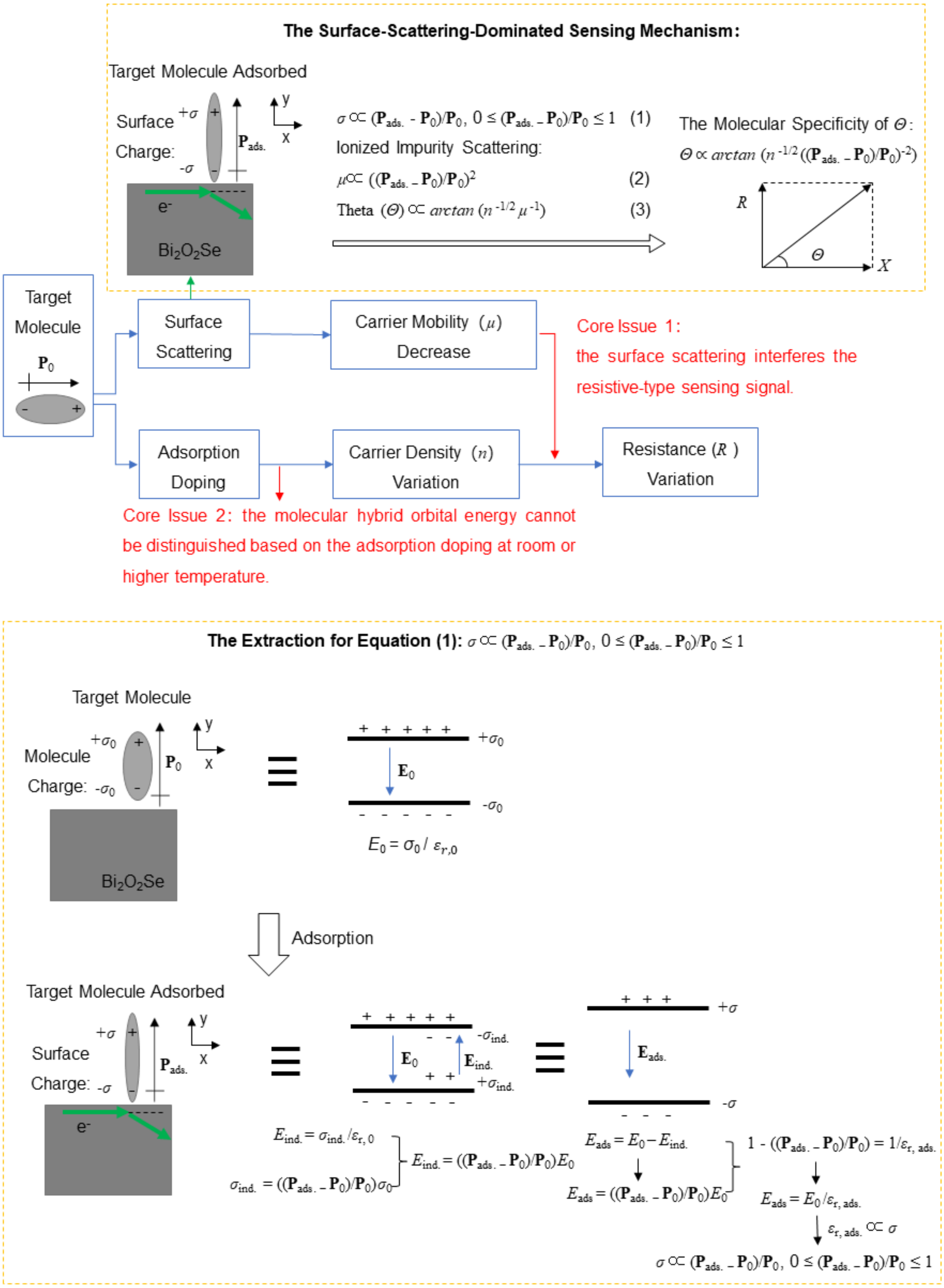

Supplementary Fig. 6| The schematic illustrating the surface-scattering-dominated sensing mechanism. Compared against the traditional mechanism (outputting the resistive signal), the surface-scattering mechanism is featured with high specificity.

Regarding the target gases, Supplementary Table 1 shows their molecular features

such as dipole moment and the highest occupied molecular orbital (HOMO) level. Muliken charge transferring is extracted for each target gas to clarify the adsorbate doping, and the surface doping can be classified into the n-type and the p-type doping. Quantum mechanical computations are based on the Gaussian 09 computational suite, obeying theory of Coupled Cluster Singles, Doubles, and Perturbative Triples; and the correlation-consistent polarized valence triple-zeta basis set is adopted for a fine balance between computational feasibility and accuracy[7-10]. Additionally, the PBE0 density functional method complemented with the D3(BJ) dispersion correction is employed to conduct all computational analyses. Established by Adamo and Barone in their foundational work on the PBE0 model, this strategy is applied for ensuring accurate and reliable density functional methods without parameter adjustment[11-12]. Noted for its precise Coulomb-fitting for elements ranging from hydrogen to radon[13], the def2-SVP basis set is utilized for atoms in the geometry optimizations phase. This basis set choice is subjected to its established accuracy and efficiency. The restricted optimizations of the crystal structure are conducted and crucial for the investigation. Unrestricted optimizations were performed for specific molecules, including nitrogen dioxide, isopropanol, formaldehyde, methanol, acetone, oxygen, ammonia, ethane, ethanol, acetic acid, formic acid, and acetaldehyde. By the unique properties and behaviors of these molecules, the distinction in methodology shows a thorough and nuanced analysis. Guided by the principles laid out in the aforementioned works, the primary research is focused on the adsorption studies on crystals and small molecules, leveraging the combination of the PBE0 density functional method and the def2-SVP basis set.

Considering that the certain value of $(\mathbf{P}_{ads.} - \mathbf{P}_0)/\mathbf{P}_0$ ($0 \leqslant (\mathbf{P}_{ads.} - \mathbf{P}_0)/\mathbf{P}_0 \leqslant 1$) is accessible for the surface-scattering-dominated sensing mechanism, the NO_2 is characterized with the highest value of $(\mathbf{P}_{ads.} - \mathbf{P}_0)/\mathbf{P}_0$ (0.07), by which the NO_2 is estimated to form a scattering center to reduce the mobility and enhance the impedance angle at the largest level.

Supplementary Table 1. The Molecular Features of Target Gas and Its Adsorbed State

Target molecule	P_0 (Debye)	$P_{ads.}$ (Debye)	$(P_{ads.} - P_0)/P_0$	HOMO (eV)	Muliken charge transferring
O_2	0	0	0	-13.16	-0.546
CH_3COOH	4.79	4.22	-0.12	-12.27	-0.045
CH_3COCH_3	3.23	2.93	-0.09	-11.18	-0.08
CH_2O	2.82	2.49	-0.12	-12.02	-0.085
CH_3OH	1.73	1.76	0.02	-12.08	0.21
CH_3CH_2OH	1.70	1.71	0.01	-12.00	0.215
CHOOH	4.38	3.97	-0.09	-12.70	-0.049
$CH_3CHOHCH_3$	1.72	1.63	-0.05	-11.86	0.223
NH_3	1.69	1.55	-0.08	-11.67	0.393
NO_2	0.45	0.48	0.07	-13.22	-0.259
CH_3CH_2CHO	3.27	2.60	-0.20	-11.40	-0.099
CH_3CH_3	0	0.32	-	-13.25	0.373

S7. The Estimation of the Electric Noise at Relatively High Frequency

To estimate the electric noise originated from the sensing response, the Fourier transform is performed on the NO_2 sensing responses involving the resistive, the reactance, and impedance angle. Herein the response variation higher than 0.02 Hz is served as electric noise since the frequency of sensing response is around 0.005 Hz. Supplementary Figure 7 shows the electric noises for the sensing response are lower than 0.4%.

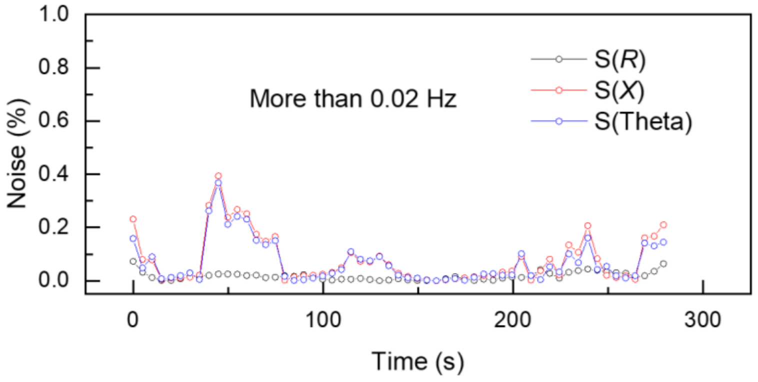

Supplementary Fig. S7| The noise of the sensing responses occurs at frequency

higher than 0.02 Hz.

S8. The structure variation for Bi_2O_2Se adsorbed with NO_2

Supplementary Figure 8 shows the structure variation for Bi_2O_2Se adsorbed with NO_2, where the quantum mechanical computations are conducted based on the Gaussian 09 computational suite. The Bi_2O_2Se remains its structure for NO_2 adsorption and the NO_2 is served as the adsorbate rather than the dopant in the Bi_2O_2Se. Supporting by the charge transferring phenomenon in Supplementary Table 1, the NO_2 doping manifests as the charge transferring from the Bi_2O_2Se to the NO_2 adsorbed, without the reconstruction of the Bi_2O_2Se.

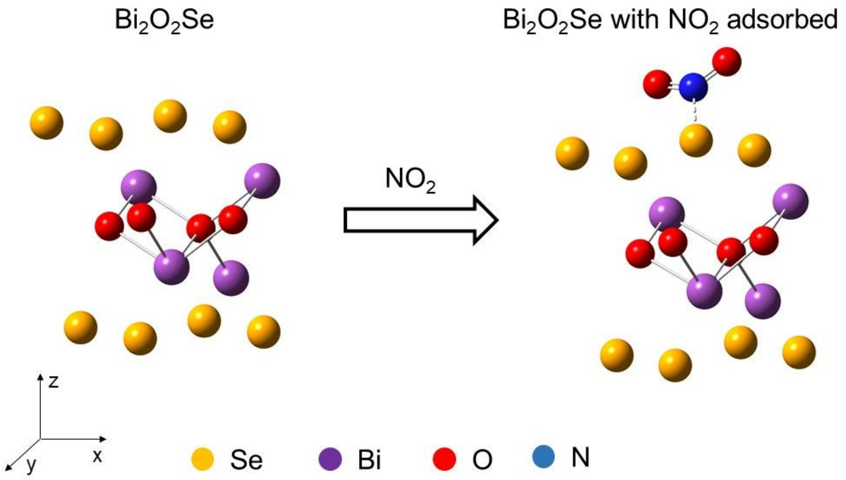

Supplementary Fig. 8| The Bi_2O_2Se structure variation for NO_2 adsorption.

S9. The Linear Fitting Data for Principle Component Analysis

Human exhale breath contains various gaseous molecules as disease markers, and their detection enables disease diagnosis with high efficiency[14-20]. NO_2 is the markers of the asthmatic, whose exhaled NO_2 concentration is hundreds of ppb higher than that of the health[18, 20, 21]. This trace NO_2 is highly active to react with the environmental oxygen. It is necessary to develop a sensing strategy with advanced features of rapid response, high specificity, and high sensitivity. This investigation develops the principle component analysis for gas sensing, aiming for the trace gas detection with molecular specificity.

For principle component analysis, this research conducts the linear fitting between the

response and the corresponding gas concentration for the sensing signals of the resistance, reactance, and impedance angle. The slope and intercept are extracted from the fitting and explored for the principle component analysis (Supplementary Table 2).

Supplementary Table 2. The Linear Fitting Data for Principle Component Analysis.

Target gas	Slop (*R*)	Intercept (*R*)	Slop (*X*)	Intercept (*X*)	Slope (*Θ*)	Intercept (*Θ*)
CH_3COCH_3	5.57×10^{-4}	8.85×10^{-4}	0.0015	0.00325	8.5×10^{-4}	0.00124
O_2	0.009	-7×10^{-4}	0.0173	4.67×10^{-4}	0.0165	-0.00167
CH_3CH_2OH	0.436	0.003	2.43	-0.0086	0.536	0.0026
CH_3CH_3	0.5	1.48	0.54	0.004	0.535	0.002
$CH_3CHOHCH_3$	0.035	0.0018	0.07	0.0066	0.04	0.003
NO_2	225	-0.05	210	-0.05	450	-0.1
NO_2/ CH_3COCH_3	160	-0.023	385	-0.06	190	-0.029
NO_2/O_2	328	-0.072	764	-0.017	397	-0.088
CHOOH	0.0025	-0.01125	0.005	-0.0135	0.0022	-0.0041
CH_3COOH	6.15×10^{-4}	-1.54×10^{-4}	0.00108	0.00523	1.85×10^{-4}	0.006
CH_2O	6.5×10^{-4}	0.0055	0.001	0.016	0.0015	0.0045
CH_3OH	1.3×10^{-5}	0.006	5.56×10^{-5}	0.0075	4.16×10^{-5}	0.00236
NH_3	0.014	-0.01	0.028	-0.018	0.016	-0.01
CH_3CH_3CHO	4.3×10^{-4}	-2.7×10^{-4}	0.00103	-0.00197	2.3×10^{-4}	2.3×10^{-4}

S10. The Correction Coefficient for Gas-concentration Calibration

To acquire the correction coefficient for gas-concentration calibration, the mass spectrometer (LINXON myRGA) is performed to analyze the partial pressures of the

gases characterized by relative molecular mass ranging from 0 to 60. The test chamber permeated with target gas (*i.e.*, acetone mixed with N_2) is of 1.1 × 10^{-4} mbar pressure. Supplementary Figure 9 shows that partial pressures of N_2 and acetone are respectively 3 × 10^{-7} mbar and 1.5 × 10^{-11}. Considering that the acetone concentration is 100 ppm mixed with N_2, the correction coefficient is estimated to be 1 × 10^{-3}. It means that the target gas will be further diluted by the other gases originated from the chamber.

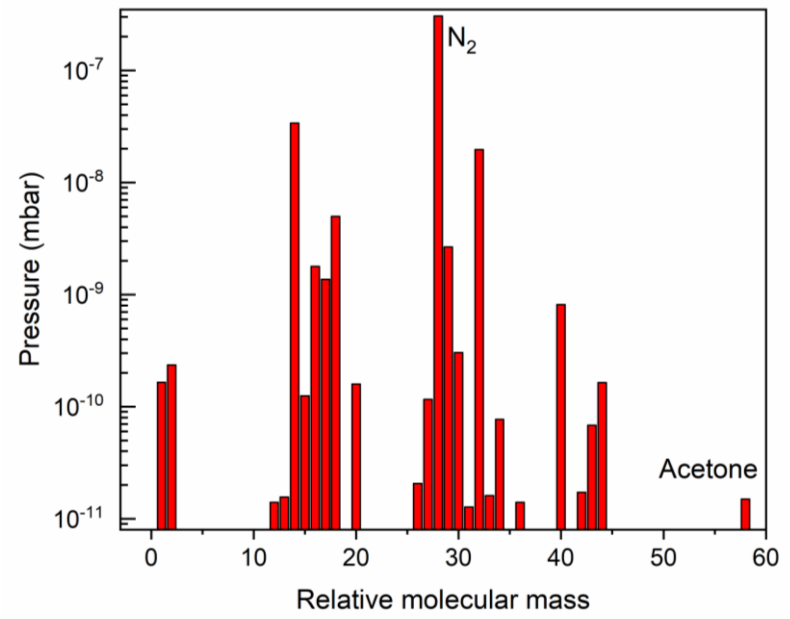

Supplementary Fig. 9| The partial pressures featured by relative molecular mass ranging from 0 to 60.

S11. The temperature-dependent *I-V* relation of the Bi_2O_2Se sensor

To further characterize the Schottky junction barrier formed along the Bi_2O_2Se-Au contact, the *I-V* relation is analyzed in a wide-range temperature ranging from 80 K to 360 K. Figure S10 presents the *I-V* curves for the sample at different temperatures (e.g., 80 K, 120 K, and 160 K), where the non-linear correlation exists for the *I-V* relation and indicates the existence of the Schottky junction barrier. To extract the Schottky barrier height, a linearly fitting between the ln (I/T^2) and T^{-1} is performed on the forward-biased contact and the slope is the value of $(qV_{ds} - \Delta E)/k_B$, according to the thermionic emission theory[22]. The inset in Supplementary Figure 10 shows that, the Schottky barrier height is extracted to be 2.952 eV, confirming the formation of

the Schottky junction barrier.

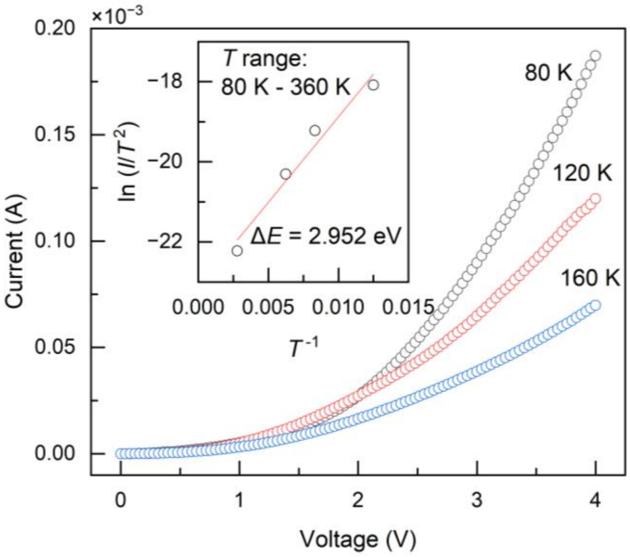

Supplementary Fig. 10| The temperature-dependent *I-V* correlation of the Bi_2O_2Se sensor and its Schottky barrier heigh analysis (the inset).

S12. The stability of the Bi_2O_2Se sensor

In Supplementary Figure 11, the Bi_2O_2Se sensor is applied for acetone detection and the original response is compared against that after 20 days. In the detection towards 7 ppm acetone after 20 days, the sensing responses remain almost constant for the multiple signals such as the resistance, the reactance, and the impedance angle. The most degradation occurs for the resistance responsivity, manifesting by around 10 % reduction as compared to the original. Regarding the reactance and the impedance angle signal, the negligible degradation exists for their responsivities. Then the sensor is ensured with the comparable stability over a long period of time.

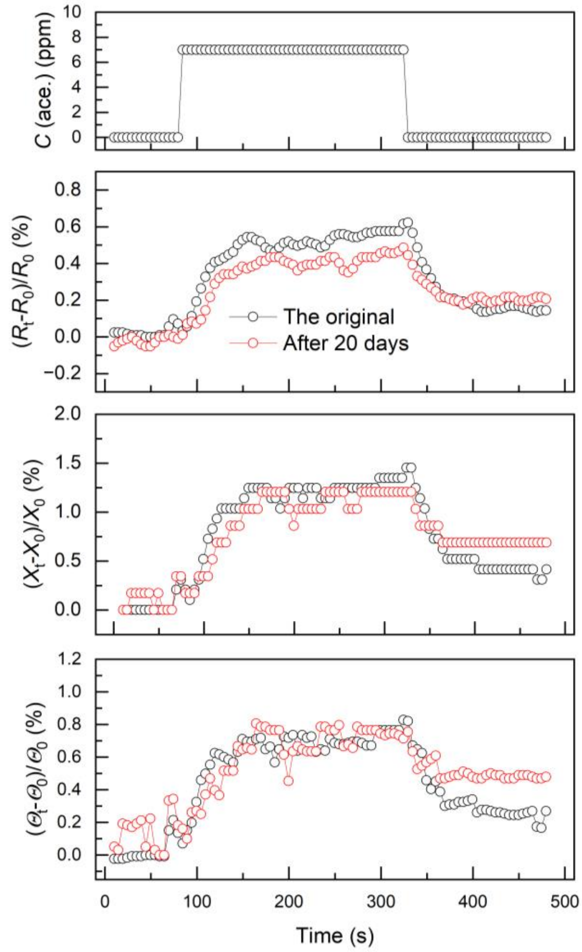

Supplementary Fig. 11| The stability test for the sensor after 20 days. The target gas is acetone (ace.) and its concentration is 7 ppm. Multiple sensing signals are collected, including the resistance, the reactance, and the impedance angle.

References

[1] Wu, J., Tan, C., Tan, Z., et al. Controlled synthesis of high-mobility atomically thin bismuth oxyselenide crystals. *Nano Lett.* **17**, 3021 (2017).

[2] Anwar, A., Nabet, B., Culp, J., Castro, F. Effects of electron confinement on thermionic emission current in a modulation doped heterostructure. *J. Appl. Phys.* **85**, 2663 (1999).

[3] Wang, J., Yao, Q., Huang, C.-W. et al. High mobility MoS_2 transistor with low schottky barrier contact by using atomic thick h-BN as a tunneling layer. *Adv. Mater.* **28**, 8302 (2016).

[4] Kohn, R.V. The relaxation of a double-well energy. *Continuum Mech.*

Thermodyn. **3**, 193 (1991).

[5] Lin, W.-Q. Li, F., Chen, G.-H., Xiao, S.-T., Wang, L.-Y., Wang, Q. A study on the adsorptions of SO_2 on pristine and phosphorus-doped silicon carbide nanotubes as potential gas sensors. *Ceramics International* **46**, 25171 (2020).

[6] Liao, N., Zheng, B., Zhang, M., Xue, W. Numerical approach to evaluate performance of porous $SiC_{5/4}O_{3/2}$ as potential high temperature hydrogen gas sensor. *Int. J. Hydrogen Energy* **44**, 26679 (2019).

[7] Lin, Y.-S., Li, G.-D., Mao, S.-P., Chai, J.-D., Chem, J. Long-range corrected hybrid density functionals with improved dispersion corrections. *Theory Comput.* **9**, 263 (2013).

[8] Weigend, F., Ahlrichs, R., Balanced basis sets of split valence, triple zeta valence and quadruple zeta valence quality for H to Rn: Design and assessment of accuracy. *Phys. Chem. Chem. Phys.* **7**, 3297 (2005).

[9] Neese, F. The ORCA program system. *WIREs Comput. Mol. Sci.* **2**, 73-78 (2012).

[10] Lu, T., Chen, F. Multiwfn: A multifunctional wavefunction analyzer. *J. Comput. Chem.* **33**, 580 (2012).

[11] Adamo, C., Barone, V. Toward reliable density functional methods without adjustable parameters: The PBE0 model. *J. Chem. Phys.* **110**, 6158-6170 (1999).

[12] Grimme, S., Antony, J., Ehrlich, S. Krieg, H. A consistent and accurate ab initio parametrization of density functional dispersion correction (DFT-D) for the 94 elements H-Pu. *J. Chem. Phys.* **132**, 154104 (2010).

[13] Weigend, F. Accurate coulomb-fitting basis sets for h to rn. *Phys. Chem. Chem. Phys.* **8**, 1057-1065 (2006).

[14] Lim, H., Kwon, H., Kang, H., Jang, J. E., Kwon, H.-J. Semiconducting MOFs on ultraviolet laser-induced graphene with a hierarchical pore architecture for NO_2 monitoring. *Nat. Commun.* **14**, 3114 (2023).

[15] van den Broek, J., Abegg, S., Pratsinis, S.E. Güntner, A.T. Highly selective detection of methanol over ethanol by a handheld gas sensor. *Nat. Commun.* **10**, 4220 (2019).

[16] Xu, S.P., Yang, C., Tian, Y., Lu, J., Jiang, Y., Guo, H.J., Zhao, J.K., Peng, H.L.

Exploitation of Schottky-junction-based sensor for specifically detecting ppt-concentration gas. *ACS Sensors* **7**, 3764 (2022).

[17] Cardenas, A., Sordillo, J.E., Rifas-Shiman, S.L. et al. The nasal methylome as a biomarker of asthma and airway inflammation in children. *Nat. Commun.* **10**, 3095 (2019).

[18] Gholizadeh, A., Voiry, D., Weisel, C., Gow, A., Laumbach, R., Kipen, H., Chhowalla, M., Javanmard, M. Toward point-of-care management of chronic respiratory conditions: Electrochemical sensing of nitrite content in exhaled breath condensate using reduced graphene oxide. *Microsystems & Nanoengineering*, **3**, 17022 (2017).

[19] Zhu, J., Ji, S., Ren, Z. Wu, W., et al. Triboelectric-induced ion mobility for artificial intelligence-enhanced mid-infrared gas spectroscopy. *Nat. Commun.* **14**, 2524 (2023).

[20] Högman, M., Lehtimäki, L., Dinh-Xuan, A. T. Utilising exhaled nitric oxide information to enhance diagnosis and therapy of respiratory disease-current evidence for clinical practice and proposals to improve the methodology. *Expert Rev. Resp. Med.* **11**, 101, (2017).

[21] Hu, Y. et al. Nitric oxide detector based on WO_3-1wt%In_2O_3-1wt%Nb_2O_5 with state-of-the-art selectivity and ppb-level sensitivity. *ACS Appl. Mater. Inter.* **10**, 42583 (2018).

[22] Sze, S.M., Li, Y., Ng, K.K. *Physics of Semiconductor Devices* (Wiley, Fourth Edition).
